# Disrupted Spatiotemporal Complexity of Resting-State Electroencephalogram Dynamics Is Associated With Adaptive and Maladaptive Rumination in Major Depressive Disorder

**DOI:** 10.3389/fnins.2022.829755

**Published:** 2022-05-09

**Authors:** Jing Wang, Qi Liu, Feng Tian, Shuzhe Zhou, Mario Alfredo Parra, Huali Wang, Xin Yu

**Affiliations:** ^1^Peking University Sixth Hospital (Institute of Mental Health), National Clinical Research Center for Mental Disorders, NHC Key Laboratory of Mental Health, Peking University, Beijing, China; ^2^Department of Psychiatry, The Second Hospital of Shanxi Medical University, Taiyuan, China; ^3^School of Psychological Sciences and Health, Department of Psychology, University of Strathclyde, Glasgow, United Kingdom

**Keywords:** major depressive disorder (MDD), complexity, rumination, oscillation, electroencephalography

## Abstract

Patients with major depressive disorder (MDD) exhibit abnormal rumination, including both adaptive and maladaptive forms. However, the neural substrates of rumination in depression remain poorly understood. We hypothesize that divergent spatiotemporal complexity of brain oscillations would be associated with the levels of rumination in MDD. We employed the multi-scale entropy (MSE), power and phase-amplitude coupling (PAC) to estimate the complexity of rhythmic dynamics from the eye-closed high-density electroencephalographic (EEG) data in treatment-naive patients with MDD (*n* = 24) and healthy controls (*n* = 22). The depressive, brooding, and reflective subscales of the Ruminative Response Scale were assessed. MDD patients showed higher MSE in timescales finer than 5 (cluster *P* = 0.038) and gamma power (cluster *P* = 0.034), as well as lower PAC values between alpha/low beta and gamma bands (cluster *P* = 0.002- 0.021). Higher reflective rumination in MDD was region-specifically associated with the more localized EEG dynamics, including the greater MSE in scales finer than 8 (cluster *P* = 0.008), power in gamma (cluster *P* = 0.018) and PAC in low beta-gamma (cluster *P* = 0.042), as well as weaker alpha-gamma PAC (cluster *P* = 0.016- 0.029). Besides, the depressive and brooding rumination in MDD showed the lack of correlations with global long-range EEG variables. Our findings support the disturbed neural communications and point to the spatial reorganization of brain networks in a timescale-dependent migration toward local during adaptive and maladaptive rumination in MDD. These findings may provide potential implications on probing and modulating dynamic neuronal fluctuations during the rumination in depression.

## Introduction

Rumination in major depressive disorder (MDD) is characterized as a recurrent, self-reflective, and uncontrollable focus on depressed mood and its potential antecedents or repercussions (Nolen-Hoeksema et al., 2008). As a central feature of MDD, it predicts severe depressive symptoms, predicts chronicity in depressed individuals, and indicates a higher risk of depressive relapses. Accumulated evidence has highlighted that rumination is a multidimensional construct (Nolen-Hoeksema et al., 2008; Zahn et al., 2015). According to the Ruminative Responses Scale (RRS), which is a frequently used self-report measure of rumination (Nolen-Hoeksema et al., 1993), rumination includes three facets: depressive rumination (RRS-D, e.g., “How often do you think about your feelings of fatigue and achiness?,” maladaptive); brooding rumination (RRS-B, e.g., “How often do you think ‘Why do I have problems other people don’t have?”’, maladaptive); and reflective rumination (RRS-R, e.g., “How often do you analyze recent events to try to understand why you are depressed?,” adaptive). In the pursuit of resiliency, reconsidering adaptive and maladaptive rumination separately is especially necessary to acquire a more comprehensive understanding of its role in the physiopathology and clinical phenotypes of MDD.

Although neuroimaging evidence using functional magnetic resonance imaging (fMRI) (Hamilton et al., 2015; Peters et al., 2019; Jacob et al., 2020) has shown that rumination is related to alterations in activation patterns and in the functional organization of different brain areas and networks, the neural substrates of adaptive vs. maladaptive rumination in depressed and non-depressed individuals have rarely been investigated separately. One study reported that higher default mode network (DMN) dominance was associated with higher levels of rumination about depressive symptoms and with lower levels of more reflective rumination in MDD (Hamilton et al., 2011). Another study using connectome-wide search found that functional connectivity between the loci in the precuneus and the right temporo-parietal junction was positively correlated with RRS scores (depressive, brooding, reflective) in the mood and anxiety disorder group (Misaki et al., 2020). However, a shortcoming of those fMRI-based measure is that they cannot capture the dynamics of neuronal interactions, which correspond in a temporally specific manner to different spatial network configurations and different aspect of stimulus processing (Andreou et al., 2015). Critically, to our knowledge, no study has evaluated the rumination-correlated rhythmic dynamics of distributed neural assemblies, reflected in electroencephalogram (EEG) data.

The EEG signal, as the rhythmic oscillation, includes the information from time, frequency, amplitude and phase. Its temporal complexity from fine (short time increment) to coarse (long time increments) timescales, which plays a fundamental role in gating the transition or exploration between different states of neural assembly, thus reflecting plasticity and adaptability in response to stressors in an ever-changing environment (McIntosh et al., 2014), can be measured by multiscale entropy (MSE) (Farzan et al., 2017). Increased MSE at fine scales, which is associated with an increased reliance on local neuronal processing, typically reflects high frequencies, whereas increased MSE at coarse scales, which is associated with increased reliance on global processing, typically reflects low frequencies (McIntosh et al., 2014). As a relatively ubiquitous phenomenon serving for coordinating high-frequency interactions across distant neuronal assemblies (Florin and Baillet, 2015; Weaver et al., 2016), phase-amplitude coupling (PAC) between low/high-frequency bands has been recognized as being involved in memory formation, consolidation and retrieval by regulating global/local neuronal communication (Bergmann and Born, 2018). Therefore, examining the relationship between the complexity of global/local neural dynamics and rumination in MDD by combining the multidomain information including time, frequency, amplitude and phase of EEG signals would allow us to advance our understanding of the neural theory of this disorder.

Given the above evidence that rumination in both MDD patients and healthy individuals involves both maladaptive and adaptive processes, we hypothesized that divergent spatiotemporal complexity of brain rhythmic oscillations in a timescale-dependent manner would be associated with the levels of adaptive and maladaptive rumination, besides the abnormal rumination and complexity of brain rhythms in depressed individuals. Therefore, in the present study, we employed MSE, power spectrum and PAC to examine the differences in the spatiotemporal complexity of resting-state EEG dynamics in depression and computed the associations of EEG complexity with measures of adaptive and maladaptive rumination in individuals with MDD and in healthy subjects.

## Materials and Methods

### Participants

Twenty-four patients with MDD were recruited from outpatient psychiatric units at the Peking University Institute of Mental Health from September 2015 to December 2016 from the subject pool of a prospective study for MDD (Lv et al., 2016) (clinicaltrial.gov identifier NCT02023567). All depressed participants met the criteria for a diagnosis of MDD according to the DSM-IV as administered by certified psychiatrists, and based on a total 17-item Hamilton Depression Rating Scale (Hamilton, 1960) (HDRS_17_) score ≥ 14. Patient exclusion criteria included psychotic episodes, substance dependence or abuse and high suicide risk based on a Mini-International Neuropsychiatric Interview (M.I.N.I.) (Si et al., 2009) assessment, history of head injury, mental instability or severe medical illness, and resistant depression. None of the recruited subjects were taking psychotropic medications at the time of assessment.

Twenty-two right-handed healthy controls (HCs) with no history of any DSM-IV psychiatric disorder were recruited from the local community and college in this study. All the HCs underwent screening that included a medical history and the M.I.N.I. to exclude the psychiatric disorders.

The study was approved by the Ethics Committee of the Peking University Institute of Mental Health. Participants gave their written informed consent prior to participating in the study and were treated in accordance with the Declaration of Helsinki.

### Rumination Assessment

The well-validated Chinese version of the 22-item RRS (Han, 2009) described above, was employed to assess rumination. RRS items were scored on a four-point Likert scale ranging from 1 (“almost never”) to 4 (“almost always”), including three facets: RRS-D (12 items), RRS-B (5 items) and RRS-R (5 items).

### Electroencephalogram Data Collection and Preprocessing

Eye closed EEG signals were continuously collected for 6 min using a 128-channel EGI HydroCel GSN electrode net (Electrical Geodesics, Inc., Eugene, OR, United States), referenced to the vertex (Cz channel) with electrode impedance below 50 kΩ. Signals were analyzed offline with the MATLAB R2014a (MathWorks, Natick, MA, United States)-based EEGLAB toolbox^1^. EEG data were resampled at 500 Hz and bandpass filtered in the range of 0.5- 45 Hz. Channelson the neck, cheeks, and forehead were excluded as these channels mostly contributed more movement-related noise. Data from 91 channels over the scalp surface were subsequently manually inspected for conspicuous baseline drift, eye movements, muscle or any other non-physiological artifact rejection. Theartifact rejection threshold was set to 100 μV. The data were average referenced.

### Electroencephalogram Signal Processing

MSE: We used MSE (Costa et al., 2005) to measure the regularity (predictability) of EEG signals from local/fine to global/coarse temporal scales. Briefly, data were first resampled to create multiple temporal scales. For a given time series, the multiple coarse-grained time series at each scale factor (in this paper referred to as timescale) were calculated by averaging the data points within non-overlapping windows. For example, scale 1 is the original time series, scale 2 averages over 2 time points. Second, sample entropy was used to measure the degree of predictability for each time series. The algorithm was available at www.physionet.org/physiotools/mse/ (Goldberger et al., 2000), with two consecutive data points for data matching (*m* = 2) and a tolerance to noise of 0.15 (*r* = 0.15). MSE estimates were calculated as the mean across within-epoch entropy measures for timescales 1–20. MSE was determined for a 60-s continuous epoch. To examine the robustness of the result, we recalculated the MSE by reducing the data length by 1/2.

Power spectrum: To examine how MSE measures relate to frequency and amplitude content, wavelet-based power spectrum analysis with a wavelet central angle frequency of 6 (ω = 6) (Li et al., 2007) was used. Signals were divided into a series of 2-s epochs. For each epoch, power values across all electrodes were computed from 0.5 to 45 Hz, with a step size of 0.5 Hz. To examine the robustness of the result, we recalculated the power by reducing the data length by 1/2. Additionally, we computed the power using the multitaper spectrum method (Behtash Babadi, 2014; Prerau et al., 2017).

PAC: To further examine global/local neuronal communication, PAC between two oscillations of high and low frequencies was assessed as described in Canolty et al. (2006). Briefly, signals were divided into a series of 2-s epochs. For each epoch, a Hilbert transform was applied to extract the instantaneous phase of low-frequency Φ_*x*_ signals and the amplitude of high-frequency α_*y*_ signals from the complex-valued analytic signals. The modulation index (MI) was the mean of the composite signal over time. The stronger the coupling between Φ_*x*_ and α_*y*_, the greater the deviation from zero was for MI. Based on our findings of power spectrum in the high-frequency bands (mainly in gamma band) between the MDD and HC groups, we focused on examining the couplings between the phase of six frequency bands [delta (δ): 1–4 Hz, theta (θ): 4–8 Hz, low alpha (α_*low*_): 8–10 Hz, high alpha (α_*high*_): 10–13 Hz, low beta (β_*low*_): 13–20 Hz, and high beta (β_*high*_): 20–30 Hz], and the amplitude of the gamma band (γ: 30–45 Hz) with a step size of 0.5 Hz. To examine the robustness of the result, we recalculated the PAC by reducing the data length by 1/2.

### Statistics

The chi-square test was conducted to assess group differences in the gender distribution. With regard to age, years of education, HDRS score and RRS score, the non-parametric permutation test (10,000 permutations) (Wang et al., 2015) were used to compare the group differences. The Pearson correlation coefficient was computed to examine the structure of the rumination subscales in the two groups. One-tailed *t*-test was employed to compare the differences of the correlation coefficient values following the Fisher z-transform.

Regarding EEG variance, the independent sample *t*-test was used to calculate the original test statistics. For the *post hoc* test comparisons in the multidimensional dataset [channels × scales, channels × frequencies, channels × each coupling pair], a cluster-based non-parametric permutation test (Maris and Oostenveld, 2007) with FieldTrip software (Oostenveld et al., 2011) was used to correct for multiple comparisons by assigning significance statistics to the probability of the size of the clusters formed by pooling adjacent electrodes with original test statistics *P* < 0.05. Identical parameters were employed across the cluster-based permutations as follows: threshold statistics of *P* < 0.05, identical neighborhood, and 10,000 permutations using a Monte Carlo approach with cluster test statistics.

The Spearman correlation coefficient was used to examine the association between EEG dynamic features and the score of rumination subscales. Similarly, a cluster-based non-parametric permutation test was applied to correct for multiple comparisons in the correlation analyses.

## Results

### Demographic, Clinical and Rumination Characteristics

There was no significant difference in age, gender, education level between the MDD and HC groups. The MDD group showed significant high HDRS score (*P* < 0.0001) (Table 1).

**TABLE 1:** Demographics and clinical characteristics of the participants.

	MDD patients (*N* = 24)	HCs (*N* = 22)	*P*
Gender (men/women)	11/13	12/10	0.5550
Age (years)	30.33 ± 1.776	28.14 ± 1.648	0.2098
Years of education	16.67 ± 0.5201	17.14 ± 0.6252	0.2094
HDRS_17_ score	20.88 ± 1.123	0.2273 ± 0.1853	< 0.0001***
Depressive episode First-episode Recurrent	177	NA NA	
RRS total score	52.67 ± 2.160	40.95 ± 2.026	0.0003
RRS-D (%)	57.52 ± 0.7284	50.82 ± 1.207	< 0.0001***
RRS-B (%)	23.04 ± 0.562	24.03 ± 0.7018	0.1624
RRS-R (%)	19.44 ± 0.5832	25.15 ± 0.7523	< 0.0001***

HDRS_17_, the 17-item Hamilton Depression Rating Scale, RRS- the Ruminative Response Scale. NA, Not applicable, and ***P < 0.001.

As expected (Table 1), the depressed participants had higher RRS scores (*P* = 0.0003), higher percentages of RRS-D (*P* < 0.0001), and lower percentages of RRS-R (*P* < 0.0001). The further correlational analysis (Table 2) indicated that rumination in the HC group had all significant correlations among the three scales (all interscale *P_*s*_* < 0.05), whereas in the MDD group, the correlation between RRS-B and RRS-R scores was not significant. In comparison, there was a significant yet weaker correlation between RRS-D and RRS-B scores (*P* = 0.0363), as well as significant decorrelation between RRS-B and RRS-R in the MDD group.

**TABLE 2:** Groupwise correlation coefficients between rumination subscales.

	MDD patients (*N* = 24)	HCs (*N* = 22)	*P* (One-tailed)
RRS-D * RRS-B	−0.62^##^	−0.86^###^	0.0363*
RRS-D * RRS-R	−0.65^###^	−0.84^###^	0.0795
RRS-B * RRS-R	–0.19	0.45^#^	0.0162*

Significant groupwise effects appear with asterisks, *P < 0.05. Additionally, significant interscale r values in each group appear with the pound, ^#^P < 0.05, ^##^P < 0.01, and ^###^P < 0.001.

### Differences in Electroencephalogram Spatiotemporal Dynamics Between the Major Depressive Disorder and Healthy Control Groups

**FIGURE 1:**
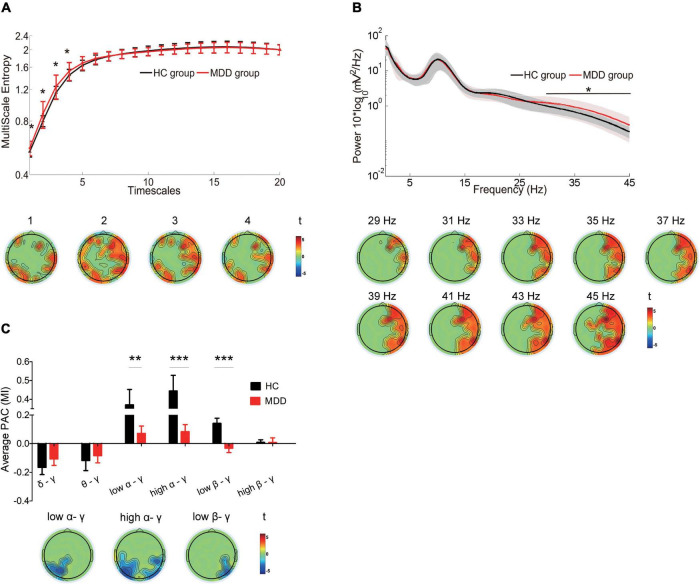
Differences in the complexity of temporal dynamics and oscillations in the MDD group compared with the complexity of temporal dynamics and oscillations of the HC group **(A)** Top: Waveforms depict MSE (y-axes) across all electrodes for timescales 1–20 (x-axes) in HCs (black line) and MDD patients (red line). Bottom: Topographic distribution of statistical significance in MSE in the MDD group compared with the HC group. Each topography reflects the significant t-maps following correction for multiple comparisons, depicting only the significant clusters with *P* < 0.05 and setting non-significant pixels to 0. Topographies highlight the spatial characteristics of the increase in MSE at fine timescales < 5 (cluster *P* = 0.038). **(B)** Top: Waveforms depict the average absolute power (y-axes) across all electrodes from 0.5 to 45 Hz (x-axes) in HCs (black line) and MDD patients (red line). Bottom: Topographic distribution of statistical significance in power in the two groups. Topographies highlight the spatial characteristics of the increase in power from 29 to 45 Hz (cluster *P* = 0.034, presented with a step of 2 Hz). **(C)** Top: Box plots depict the strength of low-frequency phase and gamma amplitude coupling in HCs (black) and MDD patients (red). The Y-axis represents the mean of the modulation index (MI) across all the electrodes. Bottom: Topographic distribution of statistical significance in PAC in the two groups. Topographies highlight the spatial characteristics of the reduction in low alpha/gamma (cluster *P* = 0.021), high alpha/gamma (cluster *P* = 0.004) and low beta/gamma (cluster *P* = 0.0017) PAC. Data were presented as mean ± SEM. Asterisks indicate significant group differences: *P < 0.05, **P < 0.01, ***P < 0.001.

In the MDD group, there was greater MSE on timescales finer than 5, increased power of high-frequency oscillations in gamma bands, and less coupling between the gamma frequency amplitude and the low alpha phase (*P* = 0.003), between the gamma frequency amplitude and the high alpha phase (*P* = 0.0004) and between the gamma frequency amplitude and the low beta phase (*P* = 0.0005) (Figures 1A–C, top). The topographic mappings in sensor space (Figure 1A, bottom) showed that the increase in MSE on fine timescales (t ranged from 1.692 to 3.102, cluster *P* = 0.038) was dispersed in the bilateral frontal, bilateral parietal regions, the right central region, the left and midline occipital region (e.g., scale factor 2). We also found that the MDD patients showed the increased power at 29–45 Hz (e.g., 35 Hz), mainly located in the right frontal, the right central, and the right parietal regions (t ranged from 1.6810 to 3.4398, cluster *P* = 0.034) (Figure 1B, bottom, presented with a step of 2 Hz). Additionally, in the MDD group, the reduced low alpha-gamma PAC was located mainly in the left parietal and occipital regions (t ranged from −3.237 to −1.9667, cluster *P* = 0.021). Reduced high alpha-gamma PAC was located mainly in the bilateral parietal and occipital regions (t ranged from −4.19 to −1.689, cluster *P* = 0.004). Decreased low beta-gamma PAC was located mainly in the right parietal and occipital regions (t ranged from −3.18 to −1.772, cluster *P* = 0.002) (Figure 1C, bottom).

In addition, the similar findings were observed after reducing the data length by 1/2, including the greater MSE on timescales finer than 5 (Supplementary Figure 1), increased gamma power (Supplementary Figure 2), and less coupling of the gamma frequency amplitude with the low alpha phase, high alpha phase and the low beta phase (Supplementary Figure 3) in the MDD group. The increased gamma power with the multitaper spectrum was consistently noticed in the MDD group (Supplementary Figure 4).

### Associations Between Electroencephalogram Spatiotemporal Dynamics and Adaptive Rumination in the Major Depressive Disorder and Healthy Control Groups

**FIGURE 2:**
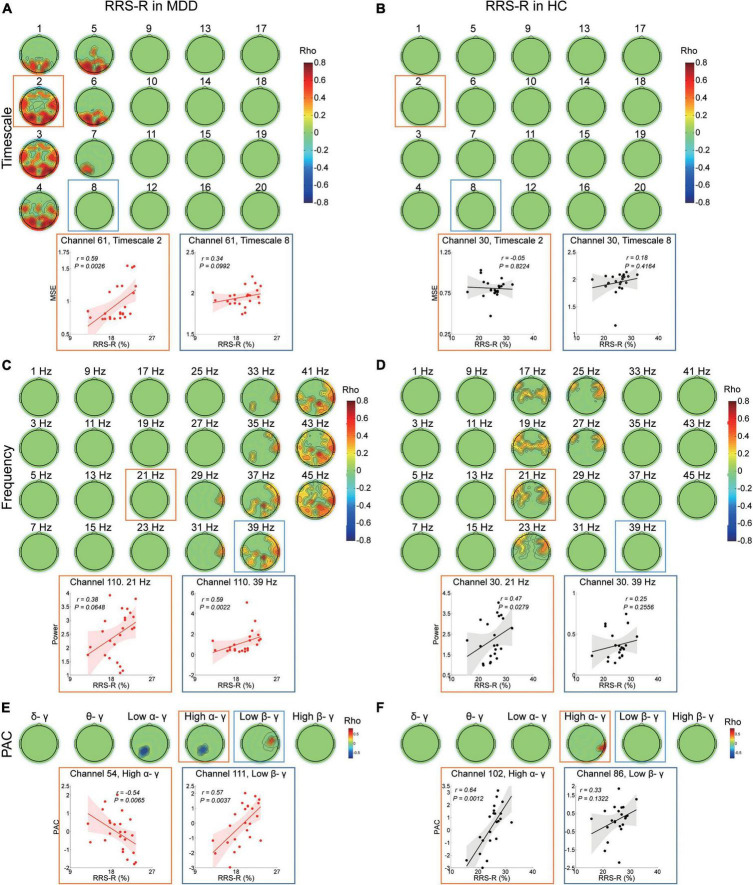
Association between EEG dynamics and adaptive rumination in the MDD and HC groups. **(A)** Top: Topographies illustrate the significant (positive cluster *P* = 0.008, cluster-based corrected) correlation between RRS-R scores and MSE in the MDD group. Bottom: Scatter plots show examples in the parietal region at fine (channel 61, timescale 2, r = 0.59, *P* = 0.003) and coarser (channel 61, timescale 8, *r* = 0.34, *P* = 0.1) timescales. **(B)** Top: Topographies illustrate no significant (cluster *P* > 0.05) correlation between the RRS-R scores and MSE in the HC group. Bottom: Scatter plots show examples in the central region at fine (channel 30, timescale 2, *r* = –0.05, *P* = 0.822) and coarser timescales (channel 30, timescale 8, *r* = 0.18, *P* = 0.416). **(C)** Top: Topographies illustrate the significant (positive clusters *P* = 0.018, cluster-based corrected) correlation between RRS-R scores and EEG power (presented with a step of 2 Hz) in the MDD group. Bottom: Scatter plots show examples in the central region in the beta (channel 110, 21 Hz, *r* = 0.38, *P* = 0.065) and gamma (channel 110, 39 Hz, *r* = 0.59, *P* = 0.002) bands. **(D)** Top: Topographies illustrate the significant (positive cluster *P* = 0.0492, cluster-based corrected) correlation between RRS-R scores and EEG power (presented with a step of 2 Hz) in the HC group. Bottom: Scatter plots show examples in the central region in the beta (channel 30, 21 Hz, *r* = 0.47, *P* = 0.028) and gamma (channel 30, 39 Hz, *r* = 0.25, *P* = 0.256) bands. **(E)** Top: Topographies illustrate the significant correlation between the PAC and RRS-R scores in the MDD group (negative cluster *P*_*low* α/γ_ = 0.016, negative cluster *P*_*high* α/γ_ = 0.029, positive cluster *P*_*low* β/γ_ = 0.042). Bottom: Scatter plots show examples of high alpha/gamma PAC in the parietal region (channel 54, *r* = –0.54, *P* = 0.007) and low beta/gamma PAC in the central region (channel 111, *r* = 0.57, *P* = 0.004). **(F)** Top: Topographies illustrate the significant correlation between PAC and the RRS-R scores in the HC group (positive cluster *P*_*high* α/γ_ = 0.037). Bottom: Scatter plots show examples of high alpha/gamma in the parietal region (channel 102, *r* = 0.64, *P* = 0.001) and low beta/gamma in the parietal region (channel 86, *r* = 0.33, *P* = 0.132) PAC.

In the MDD group, the higher RRS-R was associated with more MSE on timescales finer than 8 (r ranged from 0.409 to 0.758, cluster *P* = 0.008) (Figure 2A) at the bilateral frontal, the parietal and the occipital regions (e.g., scale factor 2). The RRS-R also showed a positive correlation with the power of high-frequency oscillations (29–45 Hz) (r ranged from 0.42 to 0.791, cluster *P* = 0.018) mainly in gamma bands (Figure 2C) across the right frontal, the right central, the bilateral parietal and the right occipital regions (e.g., 39 Hz). In addition, the higher RRS-R was correlated with greater PAC in low beta-gamma PAC (r ranged from 0.44 to 0.571, cluster *P* = 0.042) in the right central regions, and less PAC in low alpha-gamma bands (r ranged from −0.597 to −0.503, cluster *P* = 0.016) and high alpha-gamma bands (r ranged from −0.54 to −0.431, cluster *P* = 0.029) in the left parietal regions (Figure 2E).

However, in the HC group, except MSE (Figure 2B), higher RRS-R were correlated with greater power ranging from 17 to 27 Hz (r ranged from 0.405 to 0.631, cluster *P* = 0.049), mainly in beta bands (e.g., 21 Hz) at the bilateral frontal and central regions (Figure 2D), as well as greater PAC in high alpha-gamma bands (r ranged from 0.439 to 0.643, cluster *P* = 0.037) in the right parietal regions (Figure 2F).

### Associations Between Electroencephalogram Spatiotemporal Dynamics and Maladaptive Rumination in the Major Depressive Disorder and Healthy Control Groups

**FIGURE 3:**
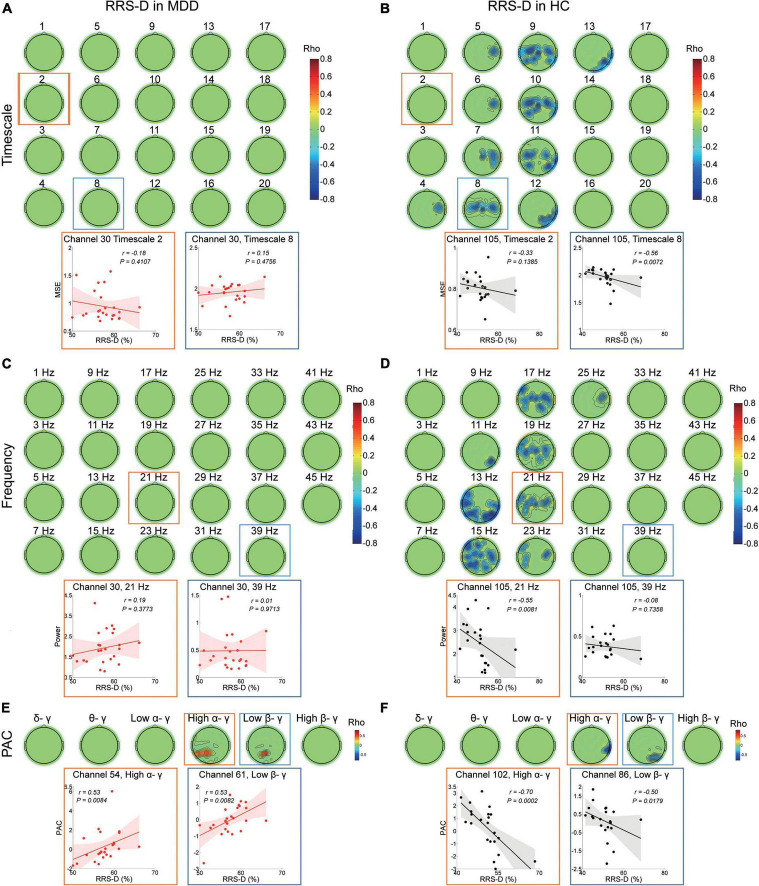
Association between EEG dynamics and depressive rumination in both the MDD and HC groups. **(A)** Top: Topographies illustrate no significant (cluster *P* > 0.05) correlation between RRS-D scores and MSE in the MDD group. Bottom: Scatter plots show examples in the central region at the fine (channel 30, timescale 2, *r* = –0.18, *P* = 0.411) and coarser timescales (channel 30, timescale 8, *r* = 0.15, *P* = 0.476). **(B)** Top: Topographies illustrate the significant correlation between the RRS-D scores and MSE in the HC group (positive cluster *P* = 0.041). Bottom: Scatter plots show examples in the central region at the fine (channel 105, Timescale 2, *r* = –0.33, *P* = 0.139) and coarser timescales (channel 105, timescale 8, *r* = –0.56, *P* = 0.007). **(C)** Top: Topographies illustrate no significant (clusters *P* > 0.05) correlation between RRS-D scores and power (presented with a step of 2 Hz) in the MDD group. Bottom: Scatter plots show examples in the central region in the beta (channel 30, 21 Hz, *r* = 0.19, *P* = 0.377) and gamma bands (channel 30, 39 Hz, *r* = 0.01, *P* = 0.971). **(D)** Top: Topographies illustrate the significant correlation (negative cluster *P* = 0.037) between the RRS-D scores and power (presented with a step of 2 Hz) in the HC group. Bottom: Scatter plots show examples in the central region in the beta (channel 105, 21 Hz, *r* = –0.55, *P* = 0.008) and gamma bands (channel 105, 39 Hz, *r* = –0.08, *P* = 0.736). **(E)** Top: Topographies illustrate the significant correlation between the RRS-D scores and PAC in the MDD group (positive cluster *P*_*high* α/γ_ = 0.007 and *P*_*low* β/γ_ = 0.022). Bottom: Scatter plots show examples of high alpha/gamma in the parietal region (channel 54, *r* = 0.53, *P* = 0.008) and low beta/gamma PAC in the parietal region (channel 61, *r* = 0.53, *P* = 0.008). **(F)** Top: Topographies illustrate the significant correlation between RRS-D scores and PAC in the HC group (negative cluster *P*_*high* α/γ_ = 0.042 and *P*_*low* β/γ_ = 0.028). Bottom: Scatter plots show examples of high alpha/gamma in the parietal region (channel 102, *r* = –0.70, *P* < 0.001) and low beta/gamma in the parietal region (channel 86, *r* = –0.50, *P* = 0.018) PAC.

In the MDD participants, the higher RRS-D was only significantly linked with greater PAC in high alpha-gamma bands (r ranged from 0.475 to 0.526, cluster *P* = 0.007) and low beta-gamma bands (r ranged from 0.527 to 0.586, cluster *P* = 0.022) in the left parietal regions (Figure 3E). There were no significant correlations with MSE from fine to coarse scales or power from 1–45 Hz (Figures 3A,C).

However, in the HC group, the higher RRS-D was associated with lower MSE on coarser timescales (4–13) (r ranged from −0.685 to −0.409, cluster *P* = 0.041) (Figure 3B) at the bilateral central and parietal regions (e.g., scale factor 8). The higher RRS-D was also correlated with weaker power ranging from 11 to 25 Hz mainly in beta bands (r ranged from −0.73 to −0.442, cluster *P* = 0.037) (Figure 3D) at the bilateral central and the left parietal regions (e.g., 21 Hz). Moreover, the level of RRS-D was negatively correlated with the strength of high alpha-gamma PAC (r ranged from −0.705 to −0.563, cluster *P* = 0.042) in the right parietal region, as well as the strength of low beta-gamma PAC (r ranged from −0.5 to −0.44, cluster *P* = 0.028) in the right parietal region (Figure 3F).

**FIGURE 4:**
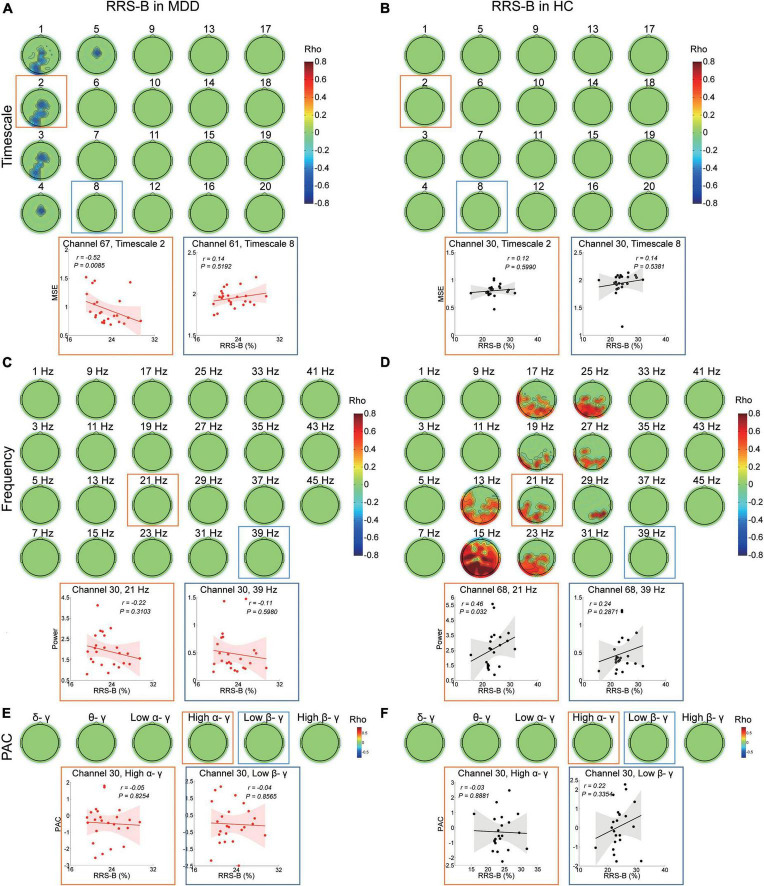
Association between EEG dynamics and brooding rumination in the MDD and HC groups. **(A)** Top: Topographies illustrate the significant correlation (negative clusters *P* = 0.048) between RRS-B scores and MSE in the MDD group. Bottom: Scatter plots show examples in the occipital region at the fine (channel 67, timescale 2, *r* = −0.52, *P* = 0.009) and in the parietal region at coarser timescales (channel 61, timescale 8, *r* = 0.14, *P* = 0.519). **(B)** Topographies illustrate no significant (clusters *P* > 0.05) correlation between RRS-B scores and MSE in the HC group. Bottom: Scatter plots show examples in the central region at the fine (channel 30, timescale 2, *r* = 0.12, *P* = 0.599) and coarser timescales (channel 30, timescale 8, *r* = 0.14, *P* = 0.538). **(C)** Topographies illustrate no significant (cluster *P* > 0.05) correlation between RRS-B scores and power (presented with a step of 2 Hz) in the MDD group. Bottom: Scatter plots show examples in the central region in the beta (channel 30, 21 Hz, *r* = −0.22, *P* = 0.31) and gamma bands (channel 30, 39 Hz, *r* = −0.11, *P* = 0.598). **(D)** Top: Topographies illustrate the significant correlation (positive cluster *P* = 0.015) between RRS-B scores and power (presented with a step of 2 Hz) in the HC group. Bottom: Scatter plots show examples in the parietal midline region in the beta (channel 68, 21 Hz, *r* = 0.46, *P* = 0.032) and gamma bands (channel 68, 39 Hz, *r* = 0.24, *P* = 0.287). **(E,F)** Top: Topographies illustrate no significant (cluster *P* > 0.05) correlation between RRS-B scores and PAC in the MDD and HC groups. Scatter plots show examples of high alpha/gamma (channel 30, *r* = −0.05, *P* = 0.825 in the MDD group; *r* = −0.03, *P* = 0.888 in the HC group) and low beta/gamma (channel 30, *r* = −0.04, *P* = 0.857 in the MDD group; *r* = 0.22, *P* = 0.335 in the HC group) PAC in the central region.

In the MDD group, RRS-B scores were only negatively associated with MSE on timescales finer than 6 (r ranged from −0.559 to −0.421, cluster *P* = 0.048) (Figure 4A) at the left occipital and midline regions (e.g., scale factor 2). There were no significant correlations with the power or PAC between gamma and lower frequencies (Figures 4C,E).

However, in the HC subjects, the level of RRS-B was only positively correlated with power ranging from 13 to 29 Hz in beta bands (r ranged from 0.373 to 0.863, cluster *P* = 0.015) (Figure 4D), primarily at the left parietal, the left occipital and parieto-occipital midline regions (e.g., 21 Hz), not with MSE or PAC (Figures 4B,F).

The physiological correlations between adaptive and maladaptive rumination and EEG variables in the MDD and HC groups discussed above are summarized in Table 3.

**TABLE 3:** Groupwise correlation summary of EEG variables and three-faceted rumination.

		RRS-R		RRS-D		RRS-B	
		MDD	HC	MDD	HC	MDD	HC
MSE							
	Timescale	Finer than 8 (+)	x	x	4–13 (−)	Finer than 6 (−)	x
	Distribution	Frontal, parietal—and occipital regions	x	x	Central and parietal regions	left occipital and midline regions	x
Power							
	Frequency	29- 45 Hz (+)	17- 27 Hz (+)	x	11–25 Hz (−)	x	13- 29 Hz (+)
	Distribution	Right hemisphere and left parietal regions	Frontal and central regions	x	Central and left parietal regions	x	Left parietal, occipital and parieto-occipital midline regions
PAC							
	Coupling pair	Low and high α-γ (−)	High α-γ (+)	High α-γ (+)	High α-γ (−)	x	x
		Low β-γ (+)	x	Low β-γ (+)	Low β-γ (−)	x	x
	Distribution	Left parietal regions	Right parietal region	Left parietal regions	Right parietal regions	x	x
		Right central regions	x	Left parietal regions	Right parietal regions	x	x

*(+), positive correlation; (−), negative correlation; x, no significant correlation. group; r = –0.03, P = 0.888 in the HC group) and low beta/gamma (channel 30, r = –0.04, P = 0.857 in the MDD group; r = 0.22, P = 0.335 in the HC group) PAC in the central region.*

## Discussion

In the present study, we examined the spatiotemporal complexities of resting-state EEG rhythms and their associations with adaptive and maladaptive rumination in major depression and healthy participants. We found abnormal rumination profiles, increased MSE on fine scales as well as enhanced gamma power and reduced PAC in depression. More interestingly, we observed different neural correlational patterns with adaptive and maladaptive rumination in MDD patients in comparison with HCs, indicating that rumination in a non-clinical state to MDD is a qualitative leap, not just the accumulation in severity.

First, patients with MDD showed more severe rumination with abnormal construct, in line with previous reports (Treynor et al., 2003; Hamilton et al., 2011). The unitary construct in HCs suggested a dynamic balance between adaptive and maladaptive rumination. However, in MDD patients, the reduced correlation between RRS-B and RRS-D scores indicates the interior disturbance within the maladaptive scales. Likewise, the lack of correlation between RRS-R and RRS-B scores probably reflected the imbalance in adaptive and maladaptive ruminations, which might cause worsening and recycling of depressive symptoms.

Second, patients with MDD showed disturbed local neural interactions and decreased global-local neural communication. The enhanced fine MSE in MDD patients indicates that local neural interactions become more complex/irregular and carry less meaningful information, reflecting decreased structural richness of brain networks (McIntosh et al., 2014). The present findings on increased gamma power, also representing disturbed local information processing in MDD patients, are in line with the phenomenon of excitatory/inhibitory (E/I) imbalance due to γ-aminobutyric acid (GABA) interneuron deficits in depression (Fee et al., 2017) and the recent insight of gamma as a biomarker for MDD (Fitzgerald and Watson, 2018). The decreased strength of cross-frequency couplings between the low alpha, high alpha, and low beta phases and gamma amplitude indicate that distributed communication at multiple spatiotemporal scales (Canolty and Knight, 2010) indeed also existed in MDD patients. Thus, our findings on resting-state EEG analysis provide further support for the imaging consensus that depression is associated with disturbed global and local brain networks (Wang et al., 2019; Yan et al., 2019) and extend previous findings from the perspective of brain complexity.

Third, and most remarkable, the adaptive reflective rumination in MDD was region-specifically associated with the more localized EEG dynamics, in comparison with those in healthy controls. We noticed that in the HC group, the enhanced beta rhythms and couplings between lower bands and gamma power might mediate flexible and informative integration during adaptive ruminative responses regarding the roles of beta bands in the formation of flexible ensembles (Lundqvist et al., 2016; Spitzer and Haegens, 2017). However, a coherent EEG temporal pattern regarding RRS-R scores in MDD was found, which reflected the reliance on local information processing, including the positive associations with the fine MSE, gamma power, and the strength of PAC between low beta and gamma bands, as well as the negative correlation with the PAC between alpha and gamma. Under the influence of enhanced complexity on fine timescales, overworked gamma activity and weakened coupling in lower band gamma, patients with MDD still tended to give rise to adaptive reflective rumination at the expense of triggering a maladaptive compensatory increase in local information processing and a decrease in the transition and communication between long-range and local neuronal encoding.

Additionally, we observed that divergent and overlapping spatial locations involved in RRS-R outcomes were present in both groups. The frontal lobes play roles in analyzing, making strategies, regulating inhibition, integrating and modifying memory and processing emotional information. The posterior brain regions such as the parietal cortex, the DMN, and the occipitotemporal regions take charge of the integration of sensory information, the integration of egocentric information, and the formation and retrieval of memories, respectively (van Wingen et al., 2010). Although these related regions were verified to subserve multidomain cognitive processing during analytical reflective responses (Berman et al., 2014; Rosenbaum et al., 2017), the differential spatial locations in the two groups might reflect the disturbance of informative processing across regions and the reorganization of networks in a pathological state.

Regarding to maladaptive rumination, the lack of correlations with long-range EEG dynamics revealed that the regression of the roles of relative long-range neural interactions on maladaptive ruminative responses in MDD patients compared with that in HCs. For instance, the RRS-D scores in HCs were negatively correlated with MSE in 4–13 scales; power in 11–25 Hz; and PAC strength among high alpha, low beta and gamma bands; these outcomes were not found for MDD patients. These results might indicate that in healthy individuals, suppressing maladaptive depressive rumination requires an increase in relatively distributed information processing and strengthens the transition and communication between long-range and local neuronal encoding. However, RRS-D scores in MDD patients were positively correlated with PAC between lower bands and gamma power, which may reflect that inhibiting depressive rumination requires weakened transition and communication between distributed and local neural activity. Regarding brooding rumination, we exclusively found a negative correlation between RRS-B scores and MSE at fine scales in MDD, which is also in line with previous reports on negative associations between functional connectivity and negative ruminative responses (Rosenbaum et al., 2017, 2018).

In addition, our observations of EEG correlates seem to be consistent with our findings on the construct of rumination subscales. In MDD patients, the stronger PAC between high alpha and gamma in the left parietal regions was linked with higher RRS-D and lower RRS-R scores, which was in line with the negative correlation between RRS-D and RRS-R scores. No overlapping spatiotemporal EEG correlates among RRS-R, RRS-D and RRS-B scores were also identical with the lack of and reduced correlation between them. Similarly, the corresponding overlapping EEG correlates were observed in accordance with all the interscale associations of rumination in HCs. Thus, the divergent spatiotemporal EEG correlates of different rumination subscales might be a possible neural explanation for the abnormal construct of rumination in depression. Moreover, it is interesting to note that the EEG features in some brain regions and temporal scales that were associated with rumination did not differentiate depressed from non-depressed individuals, supporting the formulation in which the neural system performs similar operations but carries different information (Hamilton et al., 2015).

However, several issues need to be considered further. First, the sample size was relatively small in this study, although the current sample size met the minimum requirement on the correlational analyses of the neurophysiological indexes as reported previously (Vozzi et al., 2021), future work with enlarging the samples should been done to verify the current findings. Second, the current resting-state EEG analysis only focused on the eyes closed EEG. Considering the eyes opened EEG signals have been reported to carry different information (Wu et al., 2020; Jabes et al., 2021), future work combined with both eyes open and eyes closed EEG conditions should be done. Third, although previous studies have reported that spontaneous rumination in the resting state is common in MDD patients, some bias may exist regarding the correlation between a questionnaire assessment and resting-state EEG signals. In this context, future work should explore the relationship between real-time EEG dynamics and the simultaneous ruminative mental state more explicitly. Fourth, since the study had a cross-sectional design, we could not determine whether the EEG dynamics’ change results from or confers to the abnormal rumination subscales. Using longitudinal study to provide an integrated view of the relationships between changes in the EEG dynamics and the rumination constructs may elucidate the neural pathogeny of rumination in MDD. Furthermore, we exclusively focused on the difference in adaptive and maladaptive rumination-related rhythmic oscillations, as this might provide a potential clue to identify and develop the novel neuromodulation interventions. Building models from the non-invasive or invasive electrophysiology signals to decode the rumination and mood variations (Sani et al., 2018), combining with non-invasive neuromodulation techniques such as transcranial magnetic or direct current stimulation to manipulate the regional E/I state (Hadas et al., 2019; Reinhart and Nguyen, 2019) while observing concomitant changes in neural complexity (Liang et al., 2014) might be required in future studies of precision psychiatry.

## Conclusion

The present study provides unique insights into the relationship between the intrinsic spatiotemporal reorganization of the brain and both adaptive and maladaptive rumination in depression. From the perspective of the complexity of brain oscillation dynamics, the data presented here suggest that patients with MDD have disturbed local neural processing, long-range local communications, and spatial reorganization of brain networks in a coarse/global to fine/local timescale-dependent migration during both adaptive and maladaptive ruminative information processing. These findings may provide potential directions for future research on probing and modulating dynamic global-local (coarse-fine scales) neuronal fluctuations in abnormal ruminative responses in depression.

## Data Availability Statement

The datasets presented in this study are available upon request from the corresponding author.

## Ethics Statement

The studies involving human participants were reviewed and approved by the Ethics Committee of the Peking University Institute of Mental Health. The patients/participants provided their written informed consent to participate in this study.

## Author contributions

JW contributed to the study design, performed the data analysis, and contributed to manuscript writing. QL, FT, and SZ acquired the clinical evaluation and data. MP contributed to literature review, manuscript discussion, and editing. HW and XY supervised the entire study, led the study design and literature review, and contributed to the manuscript editing. All authors have read and approved the final manuscript.

## Conflict of Interest

The authors declare that the research was conducted in the absence of any commercial or financial relationships that could be construed as a potential conflict of interest.

## Publisher’s Note

All claims expressed in this article are solely those of the authors and do not necessarily represent those of their affiliated organizations, or those of the publisher, the editors and the reviewers. Any product that may be evaluated in this article, or claim that may be made by its manufacturer, is not guaranteed or endorsed by the publisher.

## Footnotes

1
http://sccn.ucsd.edu/eeglab/

## Supplementary Material

Supplementary Figure 1 Differences in the complexity of temporal dynamics in the MDD group compared with those in the HC group. Left: Waveforms depict MSE (y-axes) across all electrodes for timescales 1–20 (x-axes) in HCs (black line) and MDD patients (red line) in the first half ofdata. *P*_*scale1*_ = 0.0634, *P*_*scale2*_ = 0.0369, *P*_*scale3*_ = 0.0677, *P*_*scale4*_ = 0.1213. Right: Waveforms depict MSE (y-axes) across all electrodes for timescales 1–20 (x-axes) in HCs (black line) and MDD patients (red line) in the second half of data. *P*_*scale1*_ = 0.0386, *P*_*scale2*_ = 0.0159, *P*_*scale3*_ = 0.0238, *P*_*scale4*_ = 0.0437. Asterisks indicate significant group differences: **P* < 0.05.

Supplementary Figure 2 Differences in the power of oscillations in the MDD group compared with those in the HC group. Left: Box plots depict the average absolute power of five frequency bands across all electrodes in HCs (black) and MDD patients (red) in the first half of data. *P*_*gamma*_ = 0.0373. Right: Box plots depict the average absolute power of five frequency bands across all electrodes in HCs (black) and MDD patients (red) in the second half of data. *P*_*gamma*_ = 0.0272. Asterisks indicate significant group differences: **P* < 0.05.

Supplementary Figure 3 Differences in the strength of low-frequency phase and gamma amplitude coupling in the MDD group compared with those in the HC group. Left: Box plots depict the strength of low-frequency phase and gamma amplitude coupling in HCs (black) and MDD patients (red) in the first half of data. The Y-axis represents the mean of the modulation index (MI) across all the electrodes. *P*_*low* α –γ_ = 0.0403, *P*_*high* α –γ_ = 0.0207, *P*_*low* β –γ_ = 0.0013. Right: Box plots depict the strength of low-frequency phase and gamma amplitude coupling in HCs (black) and MDD patients (red) in the second half of data. The Y-axis represents the mean of the modulation index (MI) across all the electrodes. *P*_δ –γ_ = 0.0461, *P*_*low* α –γ_ = 0.03, *P*_*high* α –γ_ = 0.0384, *P*_*low* β –γ_ = 0.0278. Asterisks indicate significant group differences: **P* < 0.05, ^**^*P* < 0.01.

Supplementary Figure 4 Differences in the power of oscillations in the MDD group compared with those in the HC group with the multitaper method. Box plots depict the power (dB) of five frequency bands across all electrodes in HCs (black) and MDD patients (red). *P*_*gamma*_ = 0.0062. The asterisk indicates significant group differences: ^**^*P* < 0.01.
